# Clinical and Functional Outcomes Following Primary Total Knee Arthroplasty in Patients With Knee Osteoarthritis: A Prospective Study

**DOI:** 10.7759/cureus.86944

**Published:** 2025-06-29

**Authors:** Pranav Kamlay, Rajkumar Bagewadi, Gireesh Khodnapur, Anil Bulagond, Bhimangouda S Biradar, Santosh Nandi

**Affiliations:** 1 Orthopaedics, Shri B M Patil Medical College Hospital and Research Centre, BLDE (Deemed to be University), Vijayapura, IND; 2 Orthopaedic Surgery, Shri B M Patil Medical College Hospital and Research Centre, BLDE (Deemed to be University), Vijayapura, IND

**Keywords:** knee arthroplasty, knee osteoarthritis/koa, orthopaedic surgery, primary total knee arthroplasty, total joint arthritis, total knee replacement (tkr)

## Abstract

Background

Osteoarthritis (OA) of the knee is a leading cause of disability among the elderly population, characterized by chronic pain and functional impairment. When conservative treatments fail to provide relief, total knee replacement (TKR) emerges as the gold standard surgical intervention. The effectiveness of TKR depends on appropriate patient selection, surgical technique, and structured post-operative rehabilitation.

Methods

A prospective cohort study was conducted at the Department of Orthopaedics, Shri B M Patil Medical College and Research Centre, Vijayapura, Karnataka, India, between April 2023 and December 2024. A total of 33 patients over the age of 50 years with grade 3 or 4 osteoarthritis were included after failure of non-operative therapy. All patients underwent TKR using the anteromedial parapatellar approach with a posterior-stabilized prosthesis. Patients were assessed pre-operatively and at post-operative day 5, 3 months, and six months using the Knee Society Score (KSS©). Statistical analysis was performed to evaluate score progression and its association with demographic variables.

Results

All patients had poor pre-operative KSS© (<60). By day 5 post-surgery, only 6.1% (2 out of 33) showed fair scores, while the majority remained poor. At the three-month follow-up, 72.7% (24 out of 33) of patients achieved excellent scores, and 27.2% (9 out of 33) had good scores. By six months, 100% of patients had excellent KSS©. Statistically significant associations were noted between KSS© scores at three months and both age (p=0.007) and gender (p<0.001). Complications were minimal, with only 9.1% (3 out of 33) reporting restricted range of motion and 3% (1 out of 33) experiencing superficial infection.

Conclusion

Primary TKR significantly improves clinical and functional outcomes in patients with osteoarthritic knees. Early post-operative recovery patterns may vary by age and gender, but outcomes equalize by six months. With proper surgical technique and rehabilitation, TKR proves to be a safe and highly effective treatment for end-stage knee osteoarthritis.

## Introduction

Knee osteoarthritis is a common degenerative joint disorder globally, significantly impacting the quality of life, especially in individuals over 60, and is a growing healthcare concern due to global population aging. Total knee replacement (TKR) is the gold standard surgical treatment for end-stage knee osteoarthritis, offering substantial improvements in pain, function, and quality of life. Since its introduction in the 1960s, TKR has evolved into one of the most successful orthopaedic procedures, with long-term implant survival rates exceeding 90% [1,2].

The decision to proceed with TKR is typically made when conservative management fails to provide adequate relief from symptoms. Generally, the most common underlying diagnosis associated with performing total knee arthroplasties (TKAs) across all patient age groups is primary, end-stage, and tri-compartmental osteoarthritis [3-5]. Key indications include persistent pain, significant functional limitation, and radiographic evidence of advanced joint degeneration [6]. The success of TKR depends on multiple factors, including patient selection, pre-operative planning, surgical technique, implant choice, and post-operative rehabilitation protocols. Understanding these factors and their interrelationships is crucial for optimizing outcomes and patient satisfaction.

Outcome assessment following TKR has evolved to encompass both objective clinical measures and patient-reported outcomes. Traditional evaluation methods focused primarily on implant survival and basic functional parameters. However, contemporary assessment protocols now include comprehensive evaluation of pain relief, functional recovery, range of motion, patient satisfaction, and quality of life measures [7].

Post-operative rehabilitation plays a crucial role in determining the success of TKR. Early mobilization and structured physiotherapy programs have been shown to significantly impact recovery trajectories and final outcomes [8].

Despite the overall success of TKR, complications remain a significant concern. These can range from minor issues to severe complications requiring revision surgery. Common complications include infection, instability, stiffness, and persistent pain [9]. Understanding the risk factors and developing strategies to prevent and manage these complications is essential for improving overall outcomes.

Long-term follow-up studies have demonstrated excellent durability of modern TKR implants, with survival rates exceeding 95% at 10 years [10]. However, the increasing life expectancy and higher activity levels of patients receiving TKR create new challenges regarding implant longevity and performance. This particularly affects younger patients who may require revision surgery during their lifetime.

Recent technological advances, including the use of patient-specific instrumentation and custom implants, represent potential avenues for improving outcomes. These innovations aim to optimize component positioning and restore natural knee kinematics more accurately [11,12]. Recent progress in fixation techniques for TKA has focused on optimizing implant materials and configurations to enhance bone integration, lower complication rates, and improve long-term performance. Noteworthy innovations include the use of advanced porous surfaces, such as tantalum-based trabecular metal and highly porous titanium, which facilitate improved bone ingrowth and greater implant stability. Additionally, the use of biological enhancers, including growth factors and other bioactive substances, is being investigated to accelerate integration with host bone and support long-term success of the prosthesis [13]. However, their cost-effectiveness and impact on long-term outcomes require further investigation through prospective studies.

This study evaluates short-term outcomes of TKR using the Knee Society Score (KSS©) and examines how variables like age, gender, and symptom duration may affect recovery.

## Materials and methods

This prospective cohort study was conducted at the Department of Orthopaedics, Shri B M Patil Medical College and Research Centre, Vijayapura, Karnataka, India, from April 2023 to December 2024. The study included 33 patients over 50 years of age with incapacitating knee pain due to grade 3 or 4 osteoarthritis (Kellgren & Lawrence classification) after failure of non-operative therapy.

Exclusion criteria comprised a knee with fixed flexion deformity >40%, revision TKR, patients with neurological deficit in the ipsilateral lower limb, psychological disorders, current history of septic arthritis in the same knee, valgus knee, and ipsilateral hip and ankle joint deformity and impairment.

The study was approved by the Institutional Ethics Committee, Shri B M Patil Medical College and Research Centre (Approval No. IEC/973/2022-23), and written informed consent was obtained from all participants prior to inclusion.

**Figure 1:**
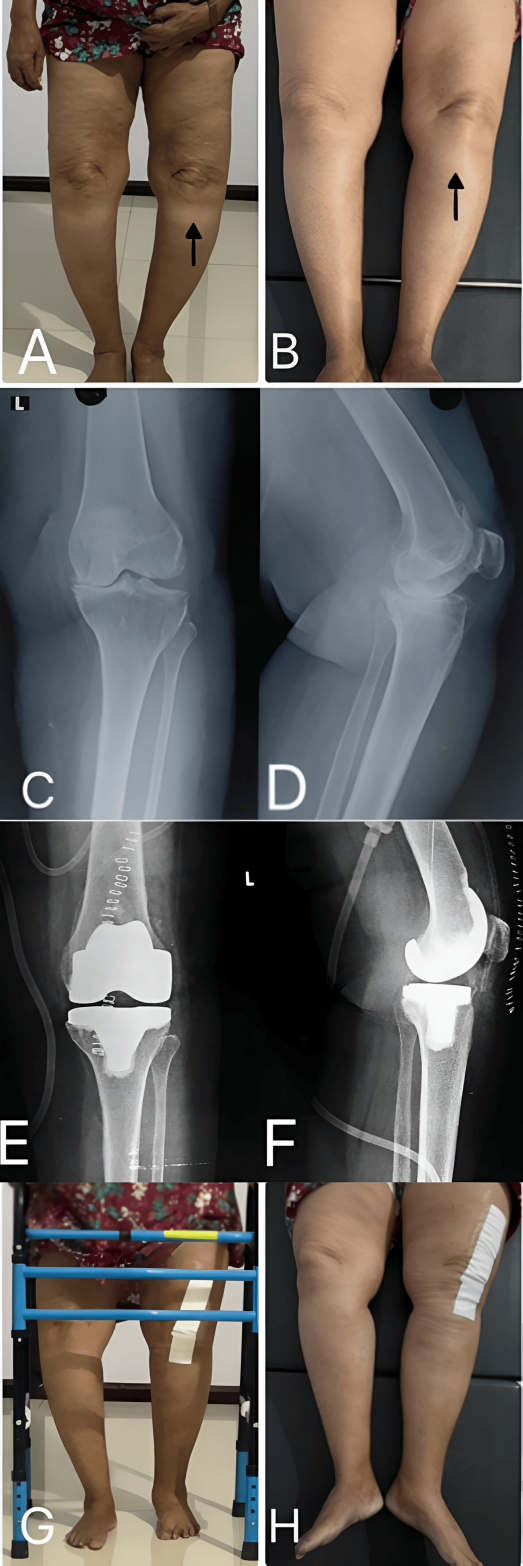
Case illustration of a 61-year-old female patient with left knee grade IV osteoarthritis (OA) (A) Clinical image of left knee osteoarthritis with varus deformity in standing position; (B) Clinical image of left knee osteoarthritis with varus deformity in supine position; (C) Pre-operative radiograph of left knee in anteroposterior (AP) view showing feature of grade IV OA knee with varus deformity; (D) Pre-operative radiograph of left knee in lateral view showing feature of grade IV OA knee; (E) Post-total knee arthroplasty radiograph of left knee in AP view with corrected varus deformity; (F) Post-total knee arthroplasty radiograph of left knee in lateral view; (G) Clinical image of patient post surgery with no varus deformity of left knee in standing position; (H) Clinical image of patient post surgery supine position

All patients underwent thorough pre-operative evaluation, including comprehensive history taking, clinical examination, and radiological assessment as described in the proforma mentioned in the appendices (see Appendix A). A standardized surgical approach was employed using the anteromedial parapatellar approach with a posterior-stabilized prosthesis design. Post-operatively, a structured rehabilitation protocol was implemented, beginning with quadriceps training exercises on the first post-operative day, followed by progressive mobilization as shown in Figure 1.

Patients were evaluated at regular intervals (pre-operative, day 5, 3rd month, and 6th month) using the KSS© [14], which assesses both objective clinical parameters and functional abilities. Each component has a maximum score of 100 points, with the results categorized as excellent (85-100), good (70-84), fair (60-69), and poor (<60) (© by The Knee Society. All rights reserved. Reproduced with permission). 

Data were analyzed using IBM SPSS Statistics for Windows, version 21 (IBM Corp., Armonk, NY, USA). Qualitative data were expressed as frequencies and percentages, while quantitative data were presented as mean, median, and standard deviation. Statistical significance was assessed using the chi-square test, Friedman test, and post hoc tests, with p<0.05 considered statistically significant.

## Results

Table 1 shows the age distribution of patients who underwent TKA, revealing that most patients were older adults, with 21.2% (7) being over 70 years of age and 48.5% (16) between 60-69 years, while middle-aged patients between <60 years constituted 30.3% (10).

**Table 1:** Distribution of patients according to age

Age (years)	No. of patients	Percentage
<60	10	30.3
60-69	16	48.5
70+	7	21.2
Total	33	100.0

Table 2 demonstrates the gender distribution among the study participants, indicating that female patients were more commonly affected by osteoarthritis requiring knee replacement, comprising 60.6% (20) of the total patients, while male patients represented 39.4% (13) of the study population.

**Table 2:** Distribution of patients according to gender

Gender	No. of patients	Percentage
Females	20	60.6
Males	13	39.4
Total	33	100.0

Table 3 presents the distribution of patients according to the affected knee side, showing a relatively balanced distribution with a slight predominance of right knee involvement at 54.5% (18) compared to left knee involvement at 45.5% (15).

**Table 3:** Distribution of patients according to the affected knee side

Affected side	No. of patients	Percentage
Left	15	45.5
Right	18	54.5
Total	33	100.0

Table 4 provides information about the duration of symptoms experienced by patients before undergoing the knee replacement surgery, with a mean duration of 29.85 months and a standard deviation (SD) of 26.279 months, suggesting considerable variation in how long patients lived with symptoms before surgical intervention.

**Table 4:** Distribution of patients according to duration of symptoms

Duration of symptoms	
Mean	29.85
Standard deviation (SD)	26.279

Table 5 illustrates the progression of KSS© at different time intervals, showing that pre-operatively, all patients (100%, 33) had poor scores, with minimal improvement by day 5 post-surgery, where 93.9% (31) still had poor scores. Only 6.1% (2) had fair scores, followed by improvement at three months when 72.7% (24) achieved excellent scores and 27.2% (9) had good scores, culminating in all patients (100%, 33) achieving excellent results in six months.

**Table 5:** Distribution of KSS© at different intervals KSS: Knee Society Score

Intervals	Poor (<60)	Fair (60-69)	Good (70-84)	Excellent (85-100)
Pre-operative	33 (100%)	-	-	-
Day 5	31 (93.9%)	2 (6.1%)	-	-
Three months	-	-	9 (27.2%)	24 (72.7%)
Six months	-	-	-	33 (100%)

Table 6 shows that among the 33 patients studied, the majority, 87.9% (29 patients), experienced no complications, while 9.1% (3 patients) developed restricted range of motion (ROM), and 3% (1 patient) had a superficial infection.

**Table 6:** Distribution of patients according to complications ROM: range of motion

Complications	Frequency	Percentage
Restricted ROM	3	9.1%
Superficial infection	1	3%
None	29	87.9%
Total	33	100%

Table 7 examines the association between age and KSS© scores at three months post-surgery, indicating that the nine patients with good (rather than excellent) scores were both in the over 60 age group, and this association was statistically significant (p=0.007).

**Table 7:** Association of KSS© at three months with age KSS: Knee Society Score p value <0.05 considered statistically significant; Chi-square test value 9.66 and degrees of freedom at 2

Age (in years)	KSS©		p-value
	Good	Excellent	
<60	0	2 (8.3%)	0.007
60-69	0	13 (54.1%)	
>70	9 (100%)	10 (41.7%)	
Total	9 (100%)	24 (100%)	

Table 8 explores the relationship between gender and KSS© at three months. KSS© at three months were rated as "excellent" in 24 patients and "good" in nine patients, with the majority "excellent" scores among female patients (79.2%, 19) compared to male patients (20.8%, 5), and this association was statistically significant (p<0.001).

**Table 8:** Association of KSS© at three months with gender KSS: Knee Society Score p value <0.05 considered statistically significant; Chi-square test value 10.01 and degrees of freedom at 1

Gender	KSS©		p-value
	Good	Excellent	
Female	1 (11.1%)	19 (79.2%)	<0.001
Male	8 (88.9%)	5 (20.8%)	
Total	9 (100%)	24 (100%)

Table 9 presents the results of a post hoc test comparing KSS© at various post-operative intervals. There was no statistically significant difference between pre-operative scores and those on POD 5 (p=0.775). However, all other comparisons (POD 5 vs. 3 months, POD 5 vs. 6 months, pre-op vs. 3 months, and pre-op vs. 6 months) showed statistically significant differences (p=0.000), indicating meaningful improvements in knee function over time.

**Table 9:** Post hoc test (pair wise comparison) KSS: Knee Society Score; POD: post-operative day p value <0.05 considered statistically significant

Pairwise comparison	Test statistics	p-value
Pre-op KSS© - POD 5	.091	.775
KSS© at POD 5 - 3 months	-1.636	.000
KSS© at POD 5 - 6 months	-2.455	.000
Pre-op KSS© - 3 months	-1.545	.000
Pre-op KSS© - 6 months	-2.364	.000

Table 10 presents the mean KSS© values obtained using the Friedman test here to compare the KSS© scores at different time points (pre-op, POD 5, 3 months, and 6 months). The F value of 87.500 indicates a high degree of variability between the groups. The P value of 0.001 is statistically significant (P < 0.05), indicating that there is a significant difference in the KSS© scores at various time points. This suggests that the post-operative changes in KSS© over time are meaningful, and the improvements observed from pre-operative to six months are not due to random chance.

**Table 10:** Pre-operative and post-operative KSS© with Friedman's ANOVA results POD: post-operative day p value<0.05 considered statistically significant

Comparison of KSS© at different follow-up intervals	KSS©		Friedman's analysis of variance	Significant value
	Mean	±SD		
Pre-op KSS©	41.197	7.5931	F=87.500	P=0.001
KSS© at POD 5	38.955	10.3012		
KSS© at 3 months	94.152	4.5043		
KSS© at 6 months	98.303	1.9801		

## Discussion

TKR represents one of the most significant surgical advancements in the management of end-stage osteoarthritis of the knee. Our prospective study aimed to evaluate the clinical and functional outcomes of primary TKR in patients with osteoarthritic knees, with special emphasis on analyzing outcomes based on demographic factors and duration of symptoms. The KSS©, a validated assessment tool, was used to objectively measure the improvement in knee function at various intervals following surgery. This discussion seeks to contextualize our findings within the broader landscape of current literature. In this study, patients who underwent TKR were above 50 years of age, with 21.2% (7) being over 70 years of age and 48.5% (16) between 60-69 years, while middle-aged patients between <60 years constituted 30.3% (10). Our findings indicated that at the three-month follow-up, 72.7% (24) of patients achieved "excellent" KSS© (85-100), while 27.2% (9) scored in the "good" range (70-84). Statistical analysis revealed a significant association between age and KSS© (p=0.007), suggesting that age may be a determinant factor for early post-operative outcomes after TKR. This observation partially aligns with Williams et al., who found that while pain relief was consistent across age groups, functional improvements were more modest in patients over 70 years [15].

The absence of a significant age-related difference in our study might be attributed to our relatively homogeneous patient selection, with strict exclusion criteria for significant comorbidities. Additionally, our standardized rehabilitation protocol, which was consistently applied across all age groups, might have mitigated potential age-related disparities in recovery. Our study demonstrated a female predominance, constituting 60.6% (20) of the study population. This gender disparity in TKR utilization is well-documented in the literature and is generally attributed to the higher prevalence of knee osteoarthritis in women. In the Indian context, studies by Pal et al. have noted that women often present later in the disease course due to social roles, limited access to care, or familial responsibilities [16]. This results in more severe deformities pre-operatively, but also provides room for greater visible functional improvement post-operatively. This is in line with a study conducted by Devasenapathy et al., where Indian women awaiting TKR exhibited significantly greater levels of disability and worse functional status compared to men at the time of surgery [17]. This disparity persisted even after accounting for differences in age, body mass index, and radiographic severity. The findings suggest that women may experience delayed access to surgical intervention or tolerate functional limitations for longer periods, underscoring the need for gender-sensitive healthcare strategies to promote earlier referral and intervention in female patients. Regarding the association between gender and TKR outcomes, our analysis showed that among patients with "good" KSS© at three months, 11.1% (1) were female individuals and 88.9% (8) were male individuals, while among those with "excellent" scores, 79.2% (19) were female individuals and 20.8% (5) were male individuals. Statistical analysis revealed a significant association between gender and KSS© (p<0.001). Our study revealed a relatively balanced distribution of affected knees, with 45.5% (15) left-sided and 54.5% (18) right-sided involvement. This slight predominance of right knee involvement is consistent with findings from larger epidemiological studies. 

Although this study focused on unilateral TKR, the high prevalence of bilateral disease suggests the need for long-term follow-up and comprehensive management. The wide variability in mean symptom duration, 29.85 months (SD 26.279), highlights the complexity of its relationship with post-operative outcomes, which remains multifaceted.

The lack of a clear association between symptom duration and post-operative outcomes in our study may be attributed to several factors. Symptom severity may be a more critical determinant of functional recovery than duration alone. Additionally, our standardized rehabilitation protocol may have mitigated the effects of prolonged symptoms. It is also possible that the KSS© does not fully capture functional deficits related to long-standing osteoarthritis.

Our study demonstrated a clear progression in KSS© from pre-operative assessment through the follow-up intervals. Pre-operatively, all patients (100%) had "poor" scores (<60), reflecting the severe functional impairment caused by end-stage osteoarthritis. The immediate post-operative assessment at day 5 showed minimal improvement, with 93.9% (31) still in the "poor" category and only 6.1% (2) improving to "fair" (60-69).

However, by the three-month follow-up, a dramatic improvement was observed, with 6.1% (2) achieving "good" scores (70-84) and the vast majority, 93.9% (31), attaining "excellent" scores (85-100). This improvement continued, with all patients (100%) achieving "excellent" scores by the six-month follow-up.

Our subgroup analysis of KSS© at three months by age categories revealed an interesting pattern. While most patients across age groups achieved "excellent" scores, all patients with "good" scores were from the >60 years category. This difference reached statistical significance (p=0.007), confirming that age-related factors affect early post-operative recovery. Similar results were reported by Chaudhary et al., who observed significant improvement in KSS© from 177 points pre-surgery to 225 points post-surgery [18]. Likewise, Navaneeth et al. observed drastic improvement in the functional ability of the patient and the ability of the patient to get back to pain-free mobilization post TKR [19].

The gender distribution in this study, 60.6% (20) female individuals, 39.4% (13) male individuals, reflects the typical epidemiological pattern of knee osteoarthritis, which disproportionately affects women. Our analysis of KSS© at three months by gender revealed that among patients with "good" scores, 11.1% (4) were female individuals and 88.9% (29) were male individuals, while among those with "excellent" scores, 79.2% (26) were female individuals and 20.8% (7) were male individuals. Statistical analysis demonstrated a significant association between gender and outcomes (p<0.001). This finding is consistent with several extensive studies in the literature.

The rapid improvement in KSS© from pre-operative values to three-month follow-up highlights the critical importance of this early recovery period. Tailored rehabilitation protocols focusing on the first three months might optimize functional outcomes.

The absence of significant outcome differences based on age, gender, or symptom duration suggests that these factors should not heavily influence patient selection for TKR.

Although our sample size of 33 patients was adequate for detecting major differences in outcomes, it may have lacked the statistical power to identify more subtle variations in subgroup analyses. This limitation could partly explain why observed differences in KSS© by age and gender did not achieve statistical significance.

The follow-up period in this study was limited to six months post-operatively. While this duration is sufficient to assess early recovery trends, it does not capture long-term outcomes such as implant survivorship, functional sustainability, or late-emerging complications.

## Conclusions

This prospective study demonstrates that TKA is a highly effective surgical intervention for end-stage osteoarthritis of the knee, resulting in significant improvements in both clinical and functional outcomes as measured by KSS©. The most notable functional gains occurred within the first three months post-operatively, underscoring the importance of early and intensive rehabilitation. Continued improvement through six months further supports the value of ongoing post-operative care. TKR outcomes were consistent across different demographics, including age, gender, and side of knee involvement, and were not significantly affected by the duration of pre-operative symptoms. This study focused on short-term outcomes up to six months post-operatively; the excellent results observed across all patient subgroups provide a strong foundation for anticipated long-term benefits. Nevertheless, continued follow-up is essential to evaluate implant durability, sustained functional improvement, and potential late complications. These findings support the use of TKR as the gold standard treatment for advanced knee osteoarthritis and highlight the importance of standardized surgical techniques, perioperative protocols, and tailored rehabilitation strategies to optimize patient outcomes. Further studies examining extended functional recovery, patient-centered outcome measures, and customized rehabilitation protocols will play a crucial role in advancing the effectiveness and consistency of care in TKA.

## Figures and Tables

**Figure 2:**
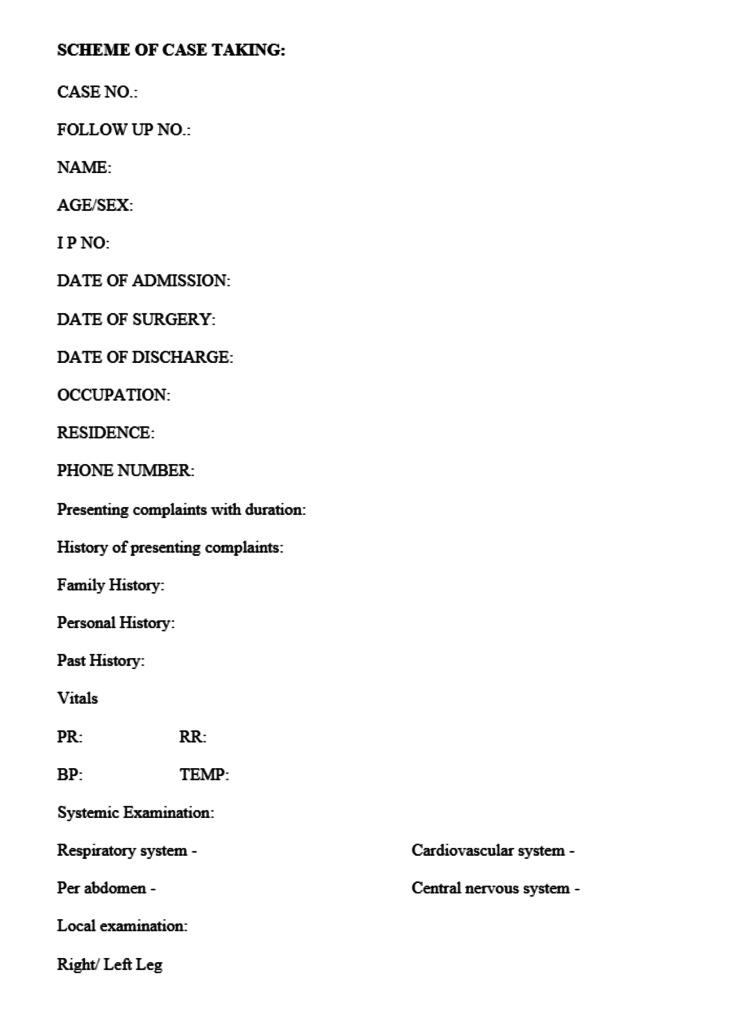
Case taking proforma, part 1 NO.: number; IP: in-patient; PR: pulse rate; RR: respiratory rate; BP: blood pressure; TEMP: temperature

**Figure 3:**
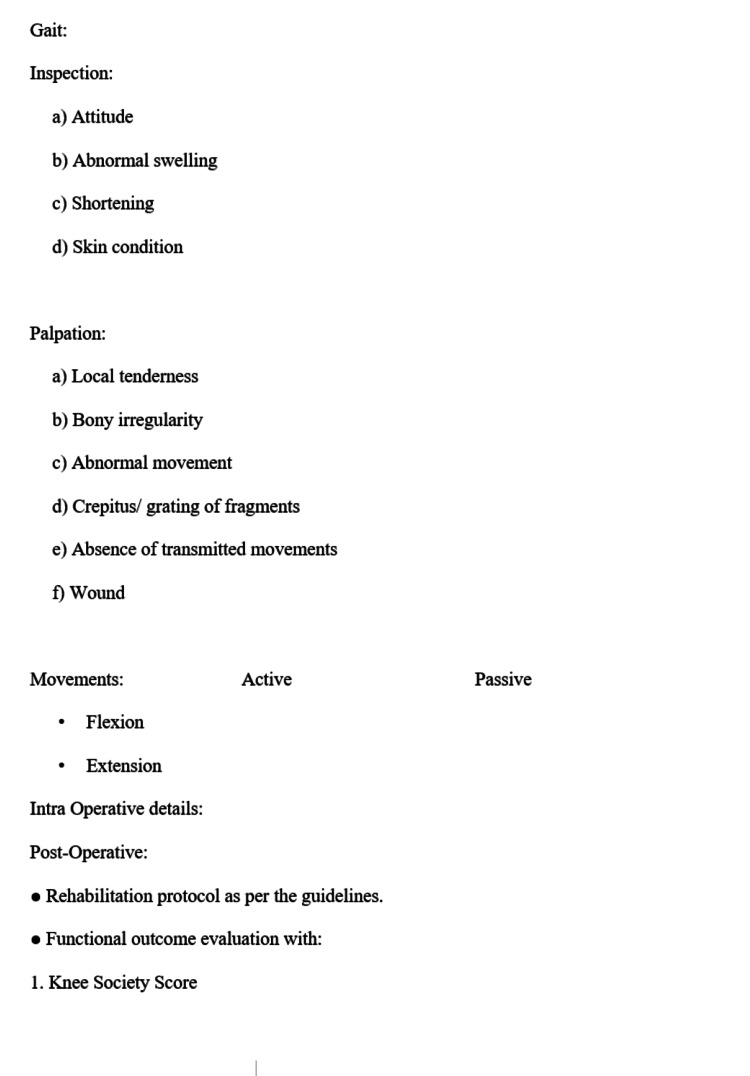
Case taking proforma, part 2

## Appendices

Appendix A

Case Taking Proforma

This structured case-taking proforma (Figures 2, 3) was developed by the author and was used to systematically collect and document patient data, including demographic details, clinical history, physical examination findings, and post-operative assessments. It served as a standardized template to ensure consistency in data acquisition throughout the study.
